# Associations between Chinese adolescents subjected to traditional and cyber bullying and suicidal ideation, self-harm and suicide attempts

**DOI:** 10.1186/s12888-019-2319-9

**Published:** 2019-10-28

**Authors:** Zhekuan Peng, Anat Brunstein Klomek, Liping Li, Xuefen Su, Lauri Sillanmäki, Roshan Chudal, Andre Sourander

**Affiliations:** 1 Injury Prevention Research Center, Shantou University Medical College, 22 Xinling Road, Shantou, 515041 China; 2 School of Psychology, Interdisciplinary Center (IDC), Herzliya, Israel; 3 Research Center for Child Psychiatry, University of Turku, Turku, Finland

**Keywords:** Bullying, Victimization, Self-harm, Suicidal ideation, Suicide attempts, Adolescents

## Abstract

**Background:**

The incidence of bullying is high among adolescents. Adolescents who were victims of bullying have a higher risk of self-harm and suicidal behavior than adolescents who were non-victims. However, research on suicide and both traditional and cyber bullying was limited in China. Therefore, this study examined the associations between Chinese adolescents who were the victims of traditional and cyber bullying and the prevalence of suicidal ideation, self-harm and suicide attempts.

**Methods:**

This was a population-based study of 2647 students (51.2% girls) with a mean age of 13.6 ± 1.1 years from 10 junior high schools in Shantou, China. Information on bullying victimization, suicidal ideation, self-harm and suicide attempts were collected using a self-administered questionnaire and the psychopathology of the students was assessed using the Strengths and Difficulties Questionnaire (SDQ). The associations were examined with multinomial logistic regression, adjusted for covariates.

**Results:**

Traditional bullying victimization was reported by 16.7% of the adolescents, cyber bullying victimization by 9.0% and both by 3.5%. The prevalence of suicidal ideation was 23.5%, self-harm was 6.2% and suicide attempts was 4.2%. Psychopathology symptoms were risk factors for suicide ideation only, ideation plus self-harm, self-harm only and suicide attempts. Victims of both traditional and cyber bullying had the highest risk of suicidal ideation only, ideation plus self-harm and suicide attempts, compared to those reporting one form of bullying. Victims of cyber bullying only had the second highest risk of suicidal ideation only and suicidal ideation plus self-harm compared to non-victims.

**Conclusions:**

Adolescents who were victims of both traditional and cyber bullying had greater risks of adverse outcomes of suicidal ideation only, suicidal ideation plus self-harm and suicide attempts. The results of the current study suggest that those exposed to both forms of bullying should be routinely screened for suicidal risk. In addition, school-based anti-bully interventions should also target cyber bullying.

## Background

Bullying is an intentional and aggressive behavior that is performed repeatedly and based on an imbalance of power between the perpetrators and the victims [1, 2]. Traditionally, bullying has been physical and verbal, which are direct forms of bullying, and relational, which is an indirect form of victimization that features exclusion and spreading rumors [3]. The rapid development of online communication, and the widespread use of instant messaging on social networking platforms, has led to the emergence of cyber bullying. This can be defined as repeated aggressive, intentional acts carried out electronically over a period of time by a group or an individual, against a victim who cannot defend themselves easily [4].

The worldwide prevalence of traditional bullying has been reported to range from 16 to 36% [5–7], whereas studies have stated that the prevalence of cyber bullying ranges from 10 to 57% [8–11] during childhood or adolescence. According to previous studies, most adolescents who experienced cyber bullying also experienced traditional bullying [12, 13]. Previous reports strongly suggest that bullying-related phenomena can differ between cultures [14]. Even in China, the prevalence of bullying and peer victimization varies from school to school [15].

Traditional and cyber bullying can have an adverse impact on the mental health of child and adolescent victims [11, 16, 17]. Both forms of bullying have been associated with depression [18], anxiety [19], low self-esteem [20], difficulties with relationships, school absenteeism [21] and substance abuse [22].

In addition, they have been reported to be associated with suicidal behavior and self-harm among adolescents [23]. Most of the studies that have been published have focused on the association between traditional bullying and suicidal risk [23–25], but some studies have also examined cyber bullying [26–29]. Studies that have quantified the risk have indicated that the harm caused by cyber bullying was comparable to traditional bullying [30, 31], whereas others have reported that cyber bullying may be even more distressing and cause greater psychological impairment than traditional school bullying [32].

To date, only a few studies have examined the associations between children and adolescents being the victims of a combination of traditional and cyber bullying, referred to as combined bullying in this paper, and suicide related outcomes. However, the results of these studies have not been consistent. On the one hand, some studies [33, 34] found that those exposed to combined bullying had the highest risk of suicidal ideation, suicidal plans, self-injury and suicide attempts. On the other hand, a longitudinal study conducted by Bannink et al. [35] found that adolescents exposed to combined bullying had no more risk of suicidal ideation than those who were the victims of either traditional or cyber bullying. These studies each included different outcomes: suicide ideation only [35], suicide ideation, suicide attempts and self-harm [34] and suicide ideation, plans, attempts and attempts requiring hospitalization [33].

To the best of our knowledge, none of the studies that have examined combined bullying have included the range of different suicide and self-injurious outcomes that can occur, while controlling for psychopathology. Moreover, studies on this issue have mainly been conducted among adolescents in Western countries. Examining the association between bullying victims and suicidal risk in China is particularly important because a lot of vicious school bullying incidents have been exposed on the Internet and people have become increasingly more aware about school bullying. Despite this, even some professionals who work with children may not always recognize bullying and understand its harmful effects. In addition, suicidality is still prevalent among adolescents in China [36].

The current study from China will add to the studies currently available from western countries. Our study included the distinct outcomes that represent various combinations of suicidality and self-harm, namely: suicidal ideation only, suicide ideation plus self-harm, self-harm only and suicide attempts.

The specific aims of the current study were three-fold. First we wanted to examine the prevalence of bullying, suicidal ideation, self-harm and suicide attempts among junior high school students in China. Our second aim was to explore the potential risk factors of suicidal ideation, self-harm and suicide attempts. Third, we aimed to examine the associations between traditional, cyber and combined bullying with suicidal ideation, self-harm and suicide attempts among the study population.

## Methods

### Study population and procedure

The study was conducted in October and November 2016 in the city of Shantou, which is located in the Eastern Guangdong Province of southern China. The research object of this study consisted of students in grades 7–9, with a mean age of 13.6 ± 1.1 years. Shantou can be divided into three areas: urban (including three districts), suburban (including three districts) and islands (including one county). Using stratified random sampling, we randomly selected 10 junior high schools and collected data from 3300 students. Five of the study sites were urban schools, three were suburban schools and two were island schools. They comprised eight government-funded schools and two private schools. The study was approved by the Ethics Committee of Shantou University Medical College and obtained permission from all the junior high schools. All the participants and their guardians agreed and provided signed, informed assent or consent on a voluntarily basis. The questionnaires were distributed to the participants by the researchers and schoolteachers and the students completed the questionnaires anonymously during a school class. The completed questionnaires were placed into an envelope, which was sealed in front of the students, and sent back to the researchers. We excluded 653 questionnaires as some of the students did not fill in the questionnaire or some questionnaires were missing too much data. In total, 2647 questionnaires were completed and included in the statistical analysis.

### Bullying victimization

The participants were asked about whether they had been bullied over the past 6 months. Traditional bullying was described in the questionnaire as: “A student is being bullied when he or she is exposed repeatedly over time to negative and hurtful actions on the part of one or more students. It is difficult for the student being bullied to defend himself or herself. Bullying may take place frequently or infrequently. Bullying can be verbal (e.g. name-calling, threats), physical (e.g. hitting), or psychological (e.g. rumors, shunning/exclusion). It is bullying when someone is teasing repeatedly in a mean or hurtful way.” We then asked the students further questions, such as how often they had been bullied at school or outside school during the past 6 months, and the possible answers were never, less than once a week, more than once a week and almost every day. The last three options were considered as being victimized at least sometimes. Cyber bullying was defined as: “when someone repeatedly makes fun of another person online or repeatedly picks on another person through e-mail or text messages or when someone posts something online about another person that they don’t like.” The participants were asked how often they had been cyber bullied during the past 6 months. The responses and the definition of sometimes were the same as for traditional bullying.

For the analysis, the participants were placed into categories based on their responses to traditional and cyber bullying victimization: 1) no form of bullying, 2) traditional bullying only, 3) cyber bullying only 4) a combination of traditional and cyber bullying.

### Suicidal ideation, self-harm and suicide attempts

Three questions concerning suicidal ideation, self-harm and suicide attempts were included in the questionnaire. Participants were asked whether they had seriously thought about committing suicide, whether they had intentionally hurt themselves by cutting or burning their skin during the past 6 months or whether they had actually tried to commit suicide. The possible answers were never, once and repeatedly and the last two options were combined as yes. As we suspected that some of the students would have simultaneously experienced suicidal ideation, self-harm and suicide attempts, we divided the participants’ responses into: 1) none of the above, 2) suicide ideation only, 3) simultaneous suicidal ideation and self-harm but not suicide attempts, 4) self-harm only, and 5) suicide attempts, regardless of whether they experienced suicidal ideation or self-harm.

### Psychopathology symptoms

The strengths and difficulties questionnaire (SDQ) [37] was used to assess the participants’ psychopathology symptoms. The SDQ is a brief behavioral screening questionnaire for 3–16 year old that comprises 25 items on psychological attributes. Each item has three options - not true, somewhat true, and certainly true - which are scored as zero, one and two respectively. These 25 items are divided into five sub-scales, with five items in each sub-scale: 1) emotional symptoms, 2) conduct problems, 3) hyperactivity/inattention, 4) peer relationship problems and 5) prosocial behavior. In this study, sub-scales one to four were used to create a total difficulties score for psychopathology symptoms with 20 items. We removed item 19 from the analysis, which related to other children or young people picking on the respondent, because it was already covered in detail in the main bullying questionnaire. Therefore, the total psychopathology scores ranged from 0 to 38.

### Statistical analysis

A descriptive analysis was performed to describe the participants’ sociodemographic characteristics and the prevalence of bullying victimization, suicidal ideation, self-harm and suicide attempts. Multinomial logistic regressions were conducted to examine the associations between traditional, cyber and combined bullying and suicidal ideation, self-harm and suicide attempts. Odds ratios (ORs) and 95% confidence intervals (95% CIs) were estimated. The gender and age of the respondent, whether the school was suburban, urban or on an island, the type of school and psychopathology were included as covariates in the logistic regression. *P* values of less than 0.05 were considered statistically significant. The data were analyzed with SPSS Statistics, version 23 (IBM Corporation, Armonk, NY, USA).

## Results

### Demographic characteristics of the participants

A total of 2647 participants participated in the study and there were slightly more girls (51.2%). The distribution of the grades, the urbanity of the school and the type of school the participants attended are shown in Table 1.

**Table 1:** Participants’ demographic characteristics and frequency distribution of behavior related to suicide and self-harm. No. (%)

Variables	Total (*n* = 2647)	Suicidal ideation, suicide attempts, and self-harm			
		Suicide attempts (*n* = 110)	Suicidal ideation plus self-harm (*n* = 79)	Suicidal ideation only (*n* = 439)	Self-harm only (*n* = 35)
Gender ^a^					
Girls	1292 (51.2)	76 (5.9)	54 (4.2)	256 (19.8)	18 (1.4)
Boys	1231 (48.8)	32 (2.6)	25 (2.0)	172 (14.0)	16 (1.3)
Grade					
7th	1269 (47.9)	53 (4.2)	35 (2.8)	217 (17.1)	20 (1.6)
8th	789 (29.8)	33 (4.2)	24 (3.0)	118 (15.0)	10 (1.3)
9th	589 (22.3)	24 (4.1)	20 (3.4)	104 (17.7)	5 (0.8)
Urbanity of school					
Urban	998 (37.7)	46 (4.6)	35 (3.5)	209 (20.9)	15 (1.5)
Suburban	1111 (42.0)	52 (4.7)	28 (2.5)	158 (14.2)	14 (1.3)
Island	538 (20.3)	12 (2.2)	16 (3.0)	72 (13.4)	6 (1.1)
Type of school					
Government-funded	2062 (77.9)	83 (4.0)	59 (2.9)	319 (15.5)	33 (1.6)
Private	585 (22.1)	27 (4.6)	20 (3.4)	120 (20.5)	2 (0.3)
Bullying victimization					
Combined bullying	92 (3.5)	13 (14.1)	7 (7.6)	33 (35.9)	0
Cyber bullying only	146 (5.5)	10 (6.8)	9 (6.2)	40 (27.4)	5 (3.4)
Traditional bullying only	350 (13.2)	20 (5.7)	14 (4.0)	70 (20.0)	9 (2.6)
Neither	2059 (77.8)	67 (3.3)	49 (2.4)	296 (14.4)	21 (1.0)

^a^ There were 124 missing data in gender

### Prevalence of bullying victimization and psychopathology symptoms

In this survey, 442 of the 2647 (16.7%) students reported being victimized by traditional bullying and 238 (9.0%) students were victims of cyber bullying. Of those, 350 (13.2%) students were victims of cyber bullying only, 146 (5.5%) students were victims of traditional bullying only and 92 (3.5%) students were victims of combined bullying (Table 1). The mean and standard deviations (SD) for the total psychopathology score of the participants was 11.3 ± 4.3.

### Prevalence of suicidal ideation, self-harm and suicide attempts

There were 621 (23.5%) students who reported suicide ideation, 165 (6.2%) who reported self-harm and 110 (4.2%) who had attempted suicide. Following the classification mentioned above, suicide ideation only was reported by 439 (16.6%) students, suicide ideation plus self-harm by 79 (3.0%) students, self-harm only by 35 (1.3%) students and suicide attempts by 110 (4.2%) students (Table 1).

### Associations between bullying victimization and suicidal ideation, self-harm and suicide attempts

In the simple logistic regression model (Table 2), traditional, cyber and combined bullying victimization were associated with suicidal ideation, self-harm and suicide attempts. There were no responses in relation to combined bullying and self-harm only and therefore that specific association was not estimated. We examined the risk posed by combined bullying, cyber bullying only and traditional bullying only. The respective risks for the three categories were: suicide ideation only (combined OR 4.6, 95% CI 2.9–7.5 versus cyber OR 2.7, 95% CI 1.8–4.0 versus traditional OR 1.6, 95% CI 1.2–2.2), suicidal ideation plus self-harm (combined OR 6.0, 95% CI 2.5–14.0 versus cyber OR 3.6, 95% CI 1.7–7.7 versus traditional OR 2.0, 95% CI 1.1–3.6) and suicide attempts (combined OR 8.1, 95% CI 4.1–15.9 versus cyber OR 3.0, 95% CI 1.5–6.0 versus traditional OR 2.0, 95% CI 1.2–3.4). The risk of cyber bullying only on self-harm was greater than that of traditional bullying only (OR 4.7, 95% CI 1.7–12.8 versus OR 2.9, 95% CI 1.3–6.5).

**Table 2:** Results of simple factor and multiple factors multinomial logistic regression of suicidal ideation, self-harm, and suicide attempts

	Suicidal ideation, suicide attempts, and self-harm OR (95% CI) ^a^			
	Suicide attempts	Suicidal ideation plus self-harm	Suicidal ideation only	Self-harm only
Simple logistic regression model				
Bullying victimization				
Combined bullying	8.1 (4.1, 15.9)***	6.0 (2.5, 14.0)***	4.6 (2.9, 7.5)***	NA
Cyber bullying only	3.0 (1.5, 6.0)**	3.6 (1.7, 7.7)**	2.7 (1.8, 4.0)***	4.7 (1.7, 12.8)**
Traditional bullying only	2.0 (1.2, 3.4)**	2.0 (1.1, 3.6)*	1.6 (1.2, 2.2)**	2.9 (1.3, 6.5)**
Neither	1.0	1.0	1.0	1.0
Multiple logistic regression model				
Psychopathology symptoms score	1.3 (1.2, 1.4)***	1.3 (1.2, 1.3)***	1.2 (1.1, 1.2)***	1.2 (1.1, 1.3)***
Gender				
Girls	2.7 (1.7, 4.3)***	2.5 (1.5, 4.2)***	1.7 (1.4, 2.2)***	1.2 (0.6, 2.4)
Boys	1.0	1.0	1.0	1.0
Grade				
7th	1.1 (0.6, 1.9)	1.0 (0.5, 1.8)	1.1 (0.8, 1.5)	2.4 (0.9, 6.5)
8th	1.1 (0.6, 2.0)	0.9 (0.5, 1.7)	0.9 (0.7, 1. 3)	1.4 (0.4, 4.3)
9th	1.0	1.0	1.0	1.0
Urbanity of school				
Urban	1.7 (0.9, 3.5)	0.9 (0.5, 1.8)	1.5 (1.1, 2.1)*	1.8 (0.6, 4.7)
Suburban	1.5 (0.8, 3.0)	0.7 (0.4, 1.3)	1.0 (0.7, 1.4)	1.3 (0.5, 3.4)
Island	1.0	1.0	1.0	1.0
Type of school				
Government-funded	0.7 (0.4, 1.2)	0.7 (0.4, 1.2)	0.7 (0.5, 0.9)**	5.0 (1.2, 21.2)*
Private	1.0	1.0	1.0	1.0
Bullying victimization				
Combined bullying	5.1 (2.3, 11.2)***	4.3 (1.7, 10.8)**	3.9 (2.3, 6.6)***	NA
Cyber bullying only	2.0 (0.9, 4.4)	2.8 (1.2, 6.1)*	2.4 (1.5, 3.6)***	4.3 (1.5, 12.1)**
Traditional bullying only	1.7 (0.9, 3.0)	1.5 (0.8, 2.9)	1.4 (1.0, 1.9)*	2.8 (1.2, 6.5)*
Neither	1.0	1.0	1.0	1.0

Abbreviation: OR, odds ratio; CI, confidence interval; NA, not applicable

^a^ The reference category for the outcome variables were none (without self-harm, suicidal ideation and suicide attempts); * *P* < 0.05, ** *P* < 0.01, *** *P* < 0.001

In the multinomial logistic regression models (Table 2), the associations between traditional, cyber and combined bullying and suicidal ideation, self-harm and suicide attempts became somewhat attenuated, after controlling for psychopathology symptoms, gender, grade, the urbanity of the school and the type of the school. Combined bullying (OR 3.9, 95% CI 2.3–6.6), cyber bullying only (OR 2.4, 95% CI 1.5–3.6) and traditional bullying only (OR 1.4, 95% CI 1.0–1.9) were significantly associated with suicidal ideation only. When it came to suicidal ideation plus self-harm, combined bullying and cyber bullying only were significant (OR 4.3, 95% CI 1.7–10.8 and OR 2.8, 95% CI 1.2–6.1). Cyber bullying only (OR 4.3, 95% CI 1.5–12.1) and traditional bullying only (OR 2.8, 95% CI 1.2–6.5) were also significantly associated with self-harm only. Combined bullying was the only category that was significantly associated with suicide attempts (OR 5.1, 95% CI 2.3–11.2).

We also compared the three types of bullying in terms of the risk of self-harm and suicide-related outcomes (Table 3). With regard to suicide ideation, those who were victims of combined bullying (OR 2.8, 95% CI 1.6–5.1) and those who were subjected to cyber bullying only (OR 1.7, 95% CI 1.1–2.9) had a higher risk than those who were the victims of traditional bullying only. Those students who were subjected to combined bullying had a higher risk of suicide ideation plus self-harm (OR 3.3, 95% CI 1.2–9.4) and suicide attempts (OR 3.2, 95% CI 1.3–8.3) compared to those who were victims of traditional bullying only.

**Table 3:** The risk of behavior related to suicide and self-harm between different types of bullying victimization

Bullying victimization ^a^	Suicidal ideation, suicide attempts, and self-harm, OR (95% CI) ^b^			
	Suicide attempts	Suicidal ideation plus self-harm	Suicidal ideation only	Self-harm only
Combined bullying vs. cyber bullying only (ref.)	2.4 (0.8, 7.2)	1.6 (0.5, 5.2)	1.7 (0.8, 3.2)	NA
Combined bullying vs. traditional bullying only (ref.)	3.2 (1.3, 8.3)*	3.3 (1.2, 9.4)*	2.8 (1.6, 5.1)**	NA
Cyber bullying only vs. traditional bullying only (ref.)	1.0 (0.4, 2.7)	1.7 (0.7, 4.4)	1.7 (1.1, 2.9)*	1.6 (0.5, 5.0)

Abbreviation: OR, odds ratio; CI, confidence interval; ref., reference; NA, not applicable

^a^ controlled the other factors: psychopathology symptoms score**,** gender, grade, urbanity of the school and the type of school

^b^ * *P* < 0.05, ** *P* < 0.01

### Other factors related to suicidal ideation, self-harm and suicide attempts

The multinomial logistic regression model showed that psychopathology symptoms, gender, the urbanity of the school and the type of the school were associated with suicidal ideation, self-harm and suicide attempts. Psychopathology symptoms were risk factors for all of the self-harm and suicidal behaviors. Girls faced a higher risk of suicidal ideation only, suicidal ideation plus self-harm and suicide attempts compared to boys. Students attending urban schools had a higher risk of suicidal ideation only compared to those from island schools. Students at local government-funded schools had a lower risk of suicidal ideation only, but a higher risk of self-harm only, compared to those at private schools.

## Discussion

The main finding of the current study was that being a victim of both traditional and cyber bullying had the strongest association with all suicide outcomes, even after controlling for baseline psychopathology. We particularly found that those who were victims of combined bullying faced the highest risk of suicide ideation only, suicidal ideation plus self-harm and suicide attempts, compared to those who were subjected to only one type of bullying. Although the students that were subjected to combined bullying accounted for the minority of the bullying victims - 3.5% of the students - they faced the highest risk of negative outcomes.

These results were in line with previous studies that found that those who experienced both traditional and cyber bullying were the most vulnerable group when it came to both emotional difficulties [38] and suicide risk [33, 39]. These results were also in line with some studies that examined the victims of various combinations of bullying and found that poly victimization was associated with a high risk of maladjustment [12] and suicidal ideation and/or behavior [40, 41]. It could also be that those who were targeted by both types of bullying felt that they did not have a safe place to escape to. This may cause unbearable mental pain [42] and lack of belonging, which increases their risk of suicidality [43]. The findings of our study are different to some previous studies, which showed that when psychopathology was controlled for, the risk for suicidality often diminished [44]. These different findings may be explained by the fact that not all studies addressed the effects of combined forms of bullying.

Our study results indicate that the second highest risk group, after those who endured combined bullying, were those who were victims of cyber bullying only, followed by those who were victims of traditional bullying only. It is important to note that 5.5% of the students in the current sample were victims of cyber bullying only. These findings were higher than a UK study [12], where just 1% of the research object were victims of cyber bullying only. Our results about the unique contribution of cyber victimization in suicide risk, over and above the contribution of traditional forms of bullying, were in line with previous studies that indicated that this form of bullying could be more harmful than traditional bullying [32]. Cyber bullying is not restricted to school grounds and hours. It can happen at any time of the day or night and be spread across a wide social network, leaving students feeling extremely isolated, dehumanized and/or helpless [4].

The prevalence of bullying in this study was lower than the rates reported by other studies [8, 17, 45] and the prevalence of self-harm was also lower than the levels reported by a previous review [46]. The prevalence of bullying in different countries around the globe may have been related to variations in cultural, religious and economic backgrounds [47, 48] and the time periods covered in various studies [17]. When we examined the association between the urbanity of the school and suicide and self-harm, the students in urban schools were at higher risk of suicidal ideation only compared to students attending the island schools. This result was different from another study in China [49], which indicated that students living in rural households had a higher risk of suicidal ideation than those living in urban households. These discrepancies can be explained by the fact that students attending the urban schools in the Shantou area may have experienced greater academic pressure than students attending island schools.

There are several limitations to the current study that need to be considered when interpreting the findings. First, the cross-sectional study design made it difficult to draw a causal association between victimization and suicidal ideation, self-harm and suicide attempts. Second, the participants completed self-reported questionnaires and the responses may have been subject to recall bias and self-reporting bias. Third, we did not consider other potential confounders, such as family or parental factors, which may have had an impact on the association between bullying victimization and suicidality. Fourth, the results about self-harm only may have limitations to extend to other junior high school students, as the sample size of self-harm only group is small.

Despite these limitations, the findings of this study have a number of important clinical and public mental health implications. Any assessments carried out on students bullying should take both traditional and cyber bullying into account. Our findings highlight the need to support victims of all forms of victimization and to pay particular attention to those who are victims of both traditional and cyber bullying, as they face the greatest risk of suicidality. These findings have considerable importance for a country like China, which has a large student population. Further research needs to be carried out on this subject and we suggest that any research should use a longitudinal multi-center design, for example including several provinces in China and adjusting for other potential cofounders, such as family or parental factors.

## Conclusions

Preventing the serious effects of cyber bullying on the quality of life of children and adolescents, and specifically the risk of suicidality, is vital given the fact that children spend increasing amounts of time in the digital world. At the same time, there is also a clear need for intervention programs to address both traditional and cyber bullying simultaneously, as this combined form of bullying poses even greater risks to young people.

## Abbreviations

CIs: Confidence intervals
ORs: Odds ratios
SD: Standard deviations
SDQ: The Strengths and Difficulties Questionnaire
